# Persistence and adherence to dutasteride/tamsulosin fixed-dose versus free-combination alpha blocker/5ARI therapy in patients with benign prostate hyperplasia in Germany 

**DOI:** 10.5414/CP203549

**Published:** 2019-10-31

**Authors:** Christoph Eisen, Zrinka Lulic, Juan Manuel Palacios-Moreno, Burkay Adalig, Michael Hennig, Vanessa Cortes, Florian Gilg, Karel Kostev

**Affiliations:** 1 GlaxoSmithKline, Munich, Germany,; 2 GlaxoSmithKline, Brentford, Middlesex, UK,; 3 GlaxoSmithKline, Madrid, Spain,; 4 GlaxoSmithKline, Istanbul, Turkey,; 5 GlaxoSmithKline, Bogotá D.C., Colombia, and; 6 IQVIA, Frankfurt, Germany; *At the time of the study

**Keywords:** persistence, adherence, benign prostatic hyperplasia, alpha blocker, 5-alpha reductase inhibitor

## Abstract

Objective: To evaluate real-world persistence and adherence in patients with benign prostate hyperplasia (BPH) receiving a fixed-dose combination of dutasteride plus tamsulosin (DUT-TAM FDC) versus α-blocker plus 5-α reductase inhibitor (AB/5ARI) free-combination therapy. Materials and methods: This retrospective, observational cohort study utilized the German IMS LRx (IQVIA) database. Patients ≥ 45 years old with BPH receiving DUT-TAM FDC or AB/5ARI free-combination therapy from July 1, 2011 to November 30, 2017 were included. Data were analyzed for 48 months from index date (date of first prescription). Persistence, measured as time to discontinuation (defined as a 90-day gap in therapy), was evaluated using Kaplan-Meier curves (log-rank tests). Adherence, measured as medication possession ratio (MPR), was based on a comparison of mean prescribing duration and expected treatment duration. Results: A total of 141,667 patients were included (DUT-TAM FDC, n = 86,057; free AB/5ARI: n = 55,610). Small differences in persistence were observed between treatment arms. At month 12, 41.8% of DUT-TAM FDC-treated and 41.0% of AB/5ARI free-combination therapy-treated patients were persistent; at month 24, 28.2% and 27.1% were persistent, respectively. A higher proportion of DUT-TAM FDC-treated patients had MPR ≥ 0.80, ≥ 0.75 and ≥ 0.70 compared with AB/5ARI free-combination therapy (p < 0.0001). Conclusion: Small differences observed in persistence between treatment arms may not translate to meaningful clinical relevance. Adherence was significantly better in the FDC arm, which may be clinically relevant as improved adherence is associated with better outcomes. Persistence and adherence to BPH therapy in Germany is low; further studies exploring the reasons behind this are required.

**What is known about this subject**

α-blocker (AB) plus 5-α reductase inhibitor (5ARI) combination therapy is superior to AB monotherapy in improving quality of life, reducing lower urinary tract symptoms, and reducing the risk of disease progression in patients with benign prostate hyperplasia (BPH).
Poor persistence and adherence to medication is common in patients with BPH, which is associated with an increased risk of complications, such as acute urinary retention and BPH-related hospitalization and surgery.
Patients with chronic diseases are more likely to adhere to a once-daily medication regimen compared with more frequent doses; therefore, medication in fixed-dose combinations, which reduce dosing frequency, may be beneficial.

**What this study adds**

This is the first study to compare real-world persistence and adherence with dutasteride-tamsulosin fixed-dose combination (DUT-TAM FDC) therapy compared with AB/5ARI in free-combination therapy in patients with BPH.
Although persistence and adherence in both treatment groups was low overall, adherence was significantly higher in patients receiving DUT-TAM FDC compared with those receiving AB/5ARI free-combination therapy. Small differences in persistence were observed between groups; however, the magnitude of differences in persistence may not translate to meaningful clinical relevance.
DUT-TAM FDC was associated with increased adherence in patients with BPH; this may be of clinical relevance to prescribers as increased adherence is associated with improved clinical outcomes, but further studies are required to investigate reasons behind low persistence and adherence overall.

## Introduction 

Benign prostatic hyperplasia (BPH) is a condition characterized by a non-malignant overgrowth of the prostate gland. It is the most common cause of lower urinary tract symptoms (LUTS) in ageing men [1, 2, 3] with ~ 1/3 of men aged 50 years or over experiencing moderate to severe LUTS [4, 5]. Due to the increase in frequency and severity with age, this condition is seen as a major public health issue with substantial associated costs [6, 7, 8]. LUTS, which may be classified as storage (irritative) symptoms, voiding (obstructive) symptoms, and/or post-micturition symptoms, have a considerable adverse impact on quality of life (QoL), psychological well-being, and interfere with activities of daily living [3, 9, 10, 11]. 

The main aim of pharmacological therapy for BPH is to improve QoL by managing urinary symptoms and preventing disease progression and complications [6]. α-blockers (AB) plus 5-α reductase inhibitors (5ARI) combination therapy is recommended as a first-line treatment for patients with BPH, with moderate to severe LUTS, at an increased risk of disease progression (i.e., those with higher prostate volume, higher prostate-specific antigen (PSA) concentration, advanced age, higher post-void residual volume, lower peak urinary flow rate (Q_max_)) [9, 12, 13] and where long-term treatment (> 12 months) is intended. Large randomized trials with a long duration of follow-up including the Medical Therapy of Prostatic Symptoms trial and the Combination of Avodart and Tamsulosin study [14] have demonstrated the superiority of AB plus 5ARI combination treatment compared with placebo or AB monotherapy. In these studies, combination therapy was shown to be effective in improving LUTS, Q_max_, QoL, and reducing the risk of disease progression [3, 14] (i.e., minimizing symptom deterioration and the need for acute urinary retention (AUR)- and BPH-related surgery) [3, 14]. More recently, the CONDUCT study demonstrated the benefit of dutasteride plus tamsulosin fixed-dose combination (DUT-TAM FDC) compared with watchful waiting (plus the initiation of tamsulosin if symptoms failed to improve), to improve symptoms and reduce the risk of disease progression [15]. 

However, consistent with many other chronic, non-life-threatening diseases, which require long-term treatment regimens, poor persistence and adherence to medication in patients with BPH is common [6, 16, 17]. Non-persistence is an independent risk factor for BPH-related hospitalization and surgery [6], and poor adherence is associated with an increased risk of AUR- and BPH-related surgery [6, 16]. In general, patients with chronic diseases are more adherent with once-daily medication regimens than more frequent doses [18, 19, 20, 21]. While most AB/5ARI combination therapy regimens require 2 – 3 tablets per day, Duodart (GlaxoSmithKline GmbH & Co. KG, Munich, Germany), a single-capsule FDC of dutasteride 0.5 mg and tamsulosin 0.4 mg launched in Germany in 2010 is conveniently administered as a 1-tablet-per-day regimen [22]. 

A recent retrospective study conducted in the Netherlands by Drake et al. [23] showed that persistence in patients receiving a FDC of solifenacin and tamsulosin for LUTS/BPH was superior to persistence in patients receiving free-combination therapy of these medications, or any combination of an anti-muscarinic and an AB. Based on these findings, DUT-TAM FDC (Duodart) may have the potential to improve persistence and adherence in patients with LUTS/BPH by reducing the frequency of dosing in comparison to AB/5ARI free-combination concomitant therapy. To date, this has not been explored and no real-world data currently exists; therefore, this study aimed to evaluate persistence and adherence to DUT-TAM FDC compared with AB/5ARI free-combination therapy in patients with LUTS/BPH in a real-world setting in Germany. 

## Materials and methods 

### Objectives 

The primary objective was to compare persistence in patients receiving DUT-TAM FDC with those receiving an AB/5ARI as free-combination therapy. Patients were followed for up to 48 months from their index date (first prescription of combination therapy). Adherence was assessed as a secondary objective. Other secondary objectives were to evaluate factors associated with persistence and adherence, and to compare prescriptions following end of initial treatment. 

### Study design 

This retrospective, observational cohort study utilized data from the IMS LRx (IQVIA) database in Germany. This database collects anonymized patient histories and patient-level prescription data from pharmacy coding centers nationwide, covering ~ 60% of all prescriptions reimbursed by statutory health insurance funds in Germany (not including private sick funds and hospital inpatient medication). Demographic data, full patient history and each prescription with full product information and prescription details is available [24]. 

### Patient population 

**Figure 1.**
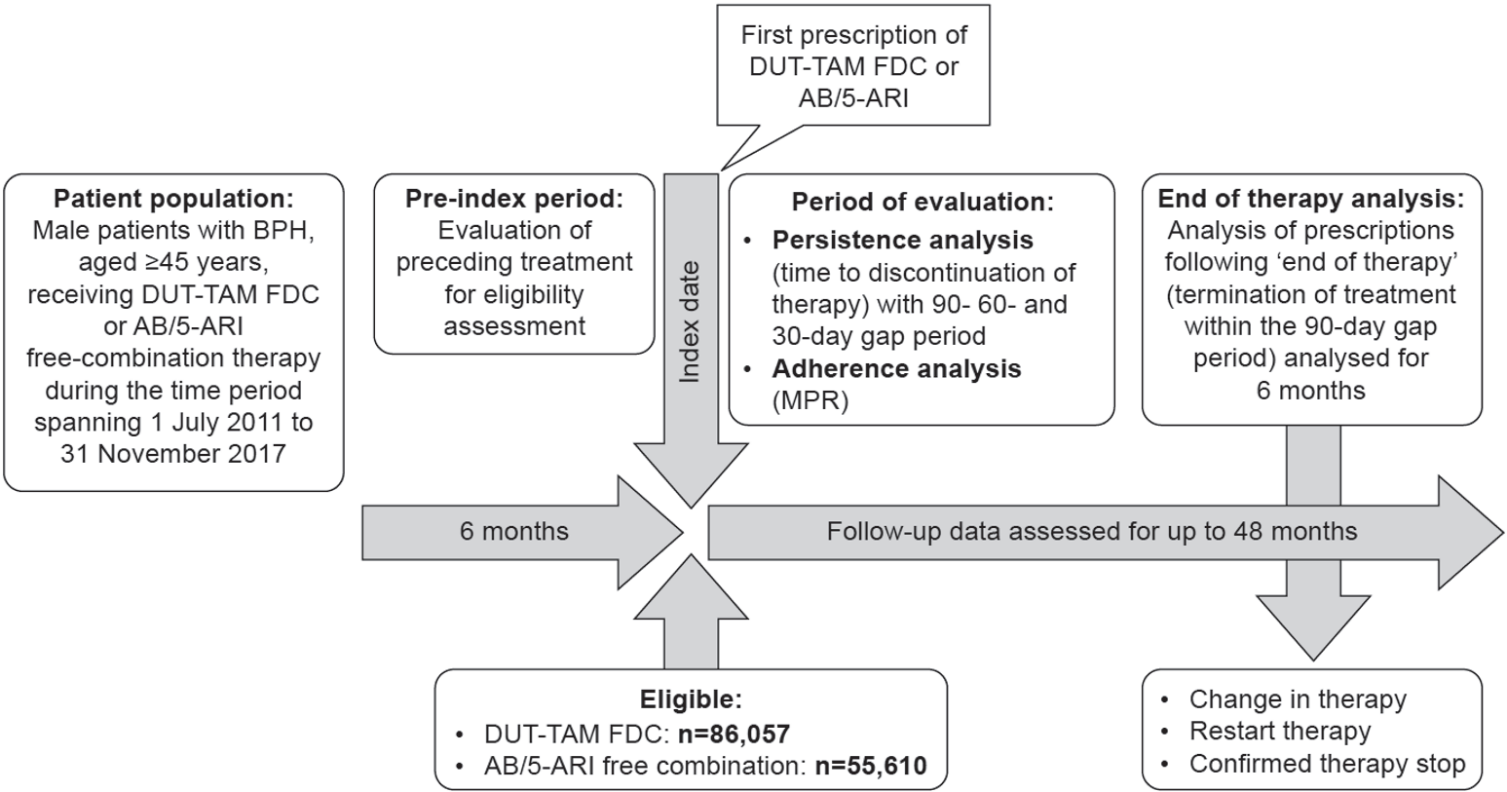
Study design. 5ARI = 5-α reductase inhibitor; AB = α-blocker; BPH = benign prostatic hyperplasia; DUT-TAM = dutasteride-tamsulosin; FDC = fixed-dose combination; MPR = medication possession ratio.

The target population were male patients with BPH, aged ≥ 45 years, receiving DUT-TAM FDC or AB/5ARI free-combination therapy (i.e., finasteride in combination with any of the following: tamsulosin, alfuzosin, terazosin, doxazosin, and silodosin) during the period spanning July 1, 2011 to November 30, 2017. For inclusion in the study, patients had to have data available for at least 6 months prior to the index date to allow evaluation of any preceding treatment. Patients who had been prescribed an AB plus 5ARI combination therapy (FDC or free combination) at any point during this pre-index date period were excluded. Follow-up data was assessed for up to 48 months from the index date (Figure 1). 

### Ethics 

Formal ethical approval from an Institutional Review Board or ethics review committee was not required for this type of observational study using anonymized data from IMS LRx. Data were anonymized at source; therefore, authors had no access to patient identifiable data. 

### Endpoints 

The primary endpoint of this study was treatment persistence, defined as the time from the index date until treatment discontinuation, in patients receiving DUT-TAM FDC versus AB/5ARI free-combination therapy. Treatment was considered discontinued when no repeat prescription had been dispensed within 90 days of the preceding therapy episode (i.e., a 90-day gap period). Available follow-up data of all patients meeting study eligibility criteria were assessed for up to 48 months from their index date for persistence. 

Time to discontinuation in patients receiving DUT-TAM FDC versus each specific AB/5ARI free-combination therapy (i.e., finasteride and each of the following: tamsulosin, alfuzosin, terazosin, doxazosin, and silodosin) was assessed as a secondary endpoint. Persistence in DUT-TAM FDC versus all concomitant usage of AB/5ARI combination therapies, including concomitant therapy with finasteride and tamsulosin was assessed. 

Secondary endpoints were evaluated by an a priori defined sensitivity analysis. Treatment persistence was assessed in patients receiving DUT-TAM FDC versus those receiving AB/5ARI free-combination therapy, with a reduced time to discontinuation of 60 and 30 days without prescription renewal (i.e. 60- and 30-day gap periods). Persistence (30- and 60-day gap period) in DUT-TAM FDC versus all concomitant usage of AB/5ARI combination therapies, and versus concomitant therapy with finasteride and tamsulosin was assessed. 

Adherence in patients receiving DUT-TAM FDC compared with AB plus 5ARI free concomitant therapy, and DUT-TAM FDC compared with concomitant therapy with finasteride and tamsulosin were assessed as further secondary endpoints. Persistence and adherence analyses were adjusted for baseline characteristics to evaluate the potential factors associated with longer persistence. Additionally, for patients in both treatment groups who discontinued DUT-TAM FDC or AB/5ARI free-combination therapy according to the 90-day gap definition, a comparison of prescriptions issued in the 6 months following treatment discontinuation was performed (end of therapy analysis). 

### Statistical analyses 

Baseline characteristics and demographics were reported descriptively. A χ^2^-test for statistical significance between groups was used for percentages and Wilcoxon tests for continuous endpoints. There was no adjustment made for multiple statistical tests. A p-value < 0.05 was considered significant. 

### Persistence and adherence 

A longitudinal dataset of medication supply was created for each patient and the number of days of drug supply calculated based on the defined daily dose. The analysis of persistence was carried out as time to discontinuation using Kaplan-Meier curves and log-rank tests (or logistic regression analyses when Kaplan-Meier curves crossed over). To identify factors affecting persistence, multivariate logistic regression models of the persistence analysis were conducted to adjust for the following baseline characteristics: age, year of therapy initiation, prescriber specialty at index date, prior treatment for BPH, previous AB treatment duration and number of concomitant drugs. Two-sided significance testing was performed. 

Adherence was measured as medication possession ratio (MPR), calculated by dividing the average prescribing distance (number of days between the index date and the first day of the last prescription) by the expected treatment duration (days) according to the therapy guidance provided in the respective prescribing information (i.e., the number of days a patient has been prescribed a medication divided by the expected duration of treatment). The proportions of patients with an MPR of ≥ 0.70, ≥ 0.75, and ≥ 0.80 were calculated and a multivariate logistic regression analysis to adjust for baseline characteristics performed (as described above for persistence) to identify any factors associated with greater adherence. 

### End-of-therapy analysis 

Patients who terminated treatment (according to the 90-day gap period definition) were assessed for any other drug prescription for an additional 6 months following therapy discontinuation and were classified as follows: change in therapy (no further prescription of index medication, but prescription of other drugs in the class, for example, AB or 5ARI monotherapy, or an alternative AB/5ARI combination, within 90 days of discontinuation), restart therapy (prescription for index medication within 90 days of discontinuation), confirmed therapy stop (no further AB and/or 5ARI prescription), or other reasons (no further prescription of any kind). 

All analyses were performed using SAS version 9.4 (SAS institute, Cary, NC, USA). All data were provided by IQVIA. 

## Results 

### Baseline characteristics 

**Figure 2.**
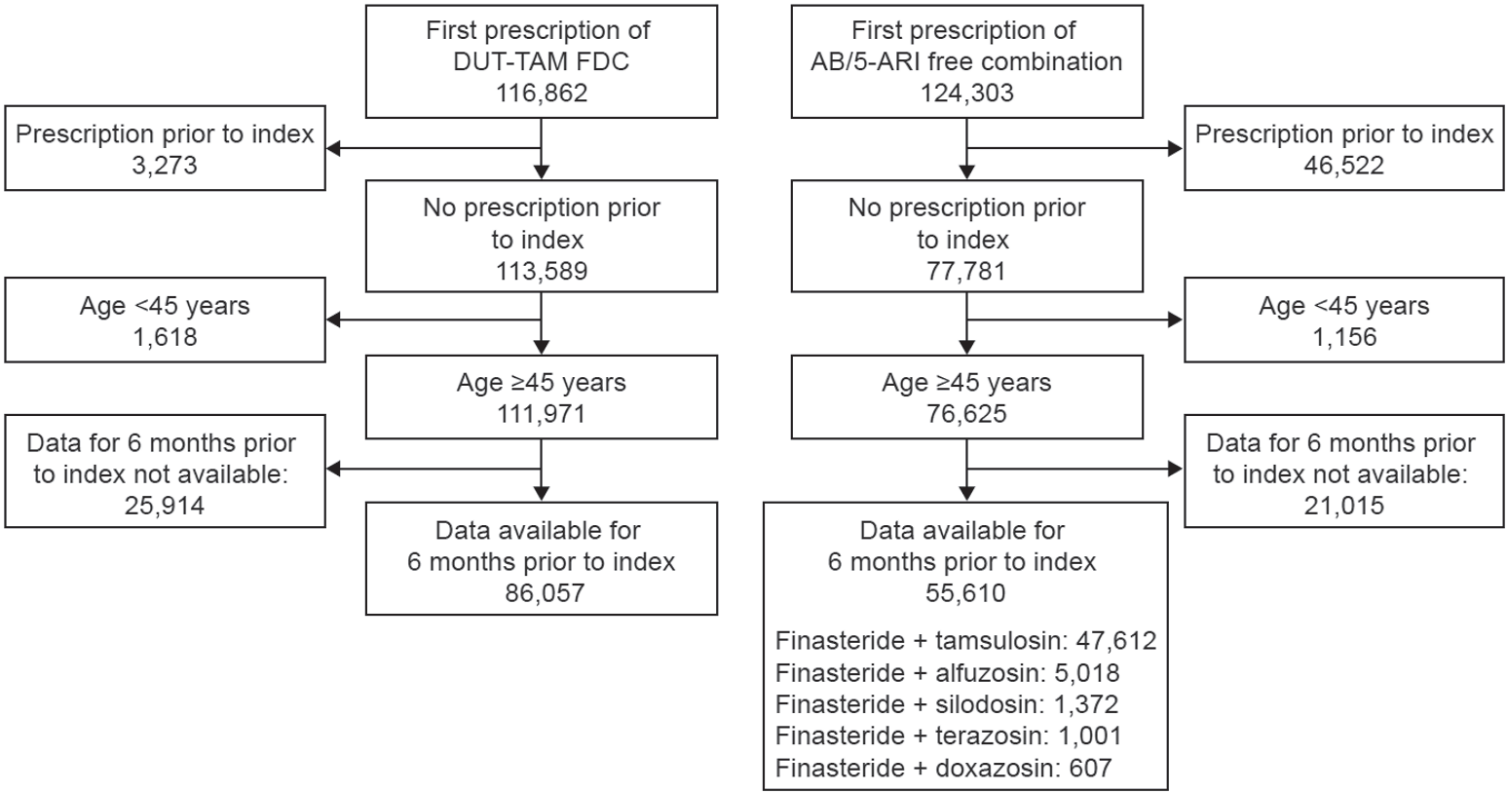
Study population. 5ARI = 5-α reductase inhibitor; AB = α-blocker; FDC = fixed-dose combination.

In total 116,862 patients received DUT-TAM FDC and 124,303 received AB/5ARI free-combination therapy during the study period, and of these, 86,057 patients treated with DUT-TAM FDC and 55,610 treated with any AB plus any 5ARI concomitant therapy were eligible for inclusion in the current analysis. The higher number of exclusions in the AB/5ARI free-combination therapy arm compared with the DUT-TAM FDC arm was likely due to the higher number of prescriptions for the index therapy prior to the index date as DUT-TAM FDC only became available on the German market in 2010 (Figure 2). 

Differences in baseline characteristics between groups were small; however, even small numerical differences were statistically significant due to the large sample size (Table 1). Tamsulosin, taken by 85.6% of patients, was the preferred AB combined with finasteride in free combination, followed by alfuzosin (9.0%), silodosin (2.5%), terazosin (1.8%), and doxazosin (1.1%). In the DUT-TAM FDC group, the percentage of patients who were therapy naïve or pre-treated with 5ARI was higher than in the AB/5ARI free-combination therapy arm, while the percentage of patients pre-treated with free AB were similar. The most common prescribers of AB/5ARI were urologists (> 80%). 

### Persistence (90-day gap definition) 

**Figure 3.**
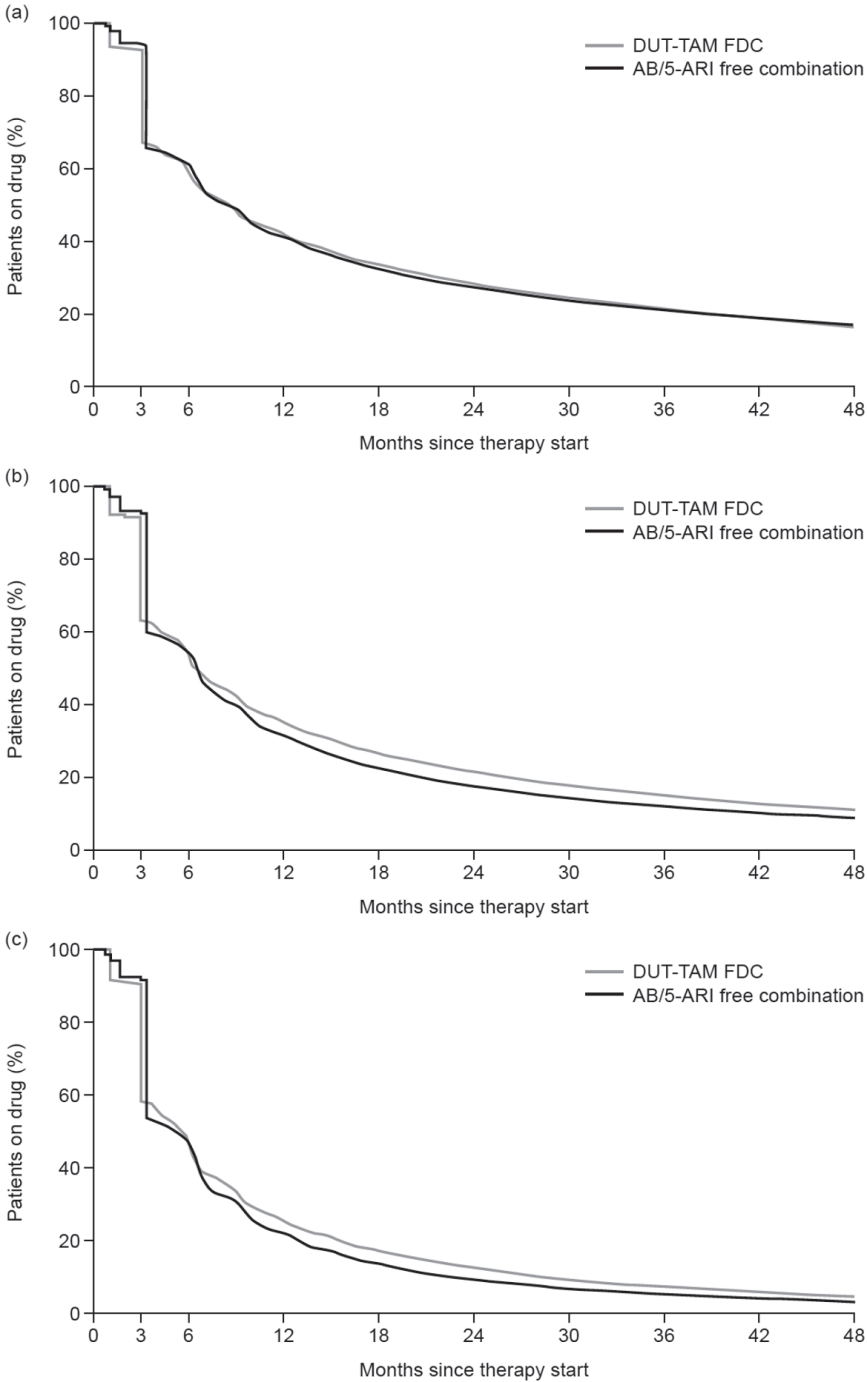
Time to discontinuation of DUT-TAM FDC vs. AB/5ARI free combination therapy for (a): the 90-day (primary endpoint), (b): 60-day, and (c): 30-day gap definitions. 5ARI = 5-α reductase inhibitor; AB = α-blocker; FDC = fixed-dose combination.

Small differences in time to discontinuation were observed between the DUT-TAM FDC group and the AB/5ARI free-combination therapy group when the 90-day gap definition was applied. Differences in persistence were in favor of AB/5ARI free-combination therapies compared with DUT-TAM FDC during the first 3 months; this was also observed at month 6, although the magnitude of difference was smaller (Figure 3A). 

There were statistically significant differences in persistence in favor of any AB/5ARI free-combination therapy at months 6 and 48. However, this significance was reversed in favor of DUT-TAM FDC at months 12, 18, and 24 versus AB/5ARI free-combination therapy (Table 2). 

Only 41.8% of DUT-TAM FDC-treated and 41.0% of AB/5ARI free-combination therapy-treated patients were persistent at month 12, which reduced to 28.2% and 27.1%, respectively, at month 24 (Table 2). 

The multivariate analysis revealed that factors associated with persistence ≥ 24 months in both groups were as follows: urologists as initial prescribers, age > 60 years, any pre-treatment, and duration of prior AB treatment exceeding 12 months (Table 3). 

### Persistence (30- and 60-day gap definitions) 

In general, persistence was low; it decreased as the gap used to define treatment discontinuation was decreased. With both 30- and 60-day gap definitions, small differences in time to discontinuation were observed between the DUT-TAM FDC- and AB/5ARI free-combination therapy groups. Differences in persistence were in favor of AB/5ARI free-combination therapies compared with DUT-TAM FDC during the first 3 months; this was also observed at month 6, although the magnitude of difference was smaller (Figure 3B, C). 

With a 60-day gap definition, logistic regression analysis demonstrated statistically significant differences in persistence in favor of AB/5ARI free-combination therapy versus DUT-TAM FDC (54.6 versus 53.6%, p < 0.0001) at month 6. Differences were statistically significant in favor of DUT-TAM FDC- versus AB/5ARI free-combination therapy and versus tamsulosin/finasteride at all other time points (months 12 – 48, all p < 0.0001). With a 30-day gap definition, logistic regression analysis demonstrated statistically significant differences in persistence in favor of DUT-TAM FDC- versus free AB/5ARI combination therapy at all time points, and versus tamsulosin/finasteride at all time points except at month 6 (all p < 0.0001). 

### Adherence (sensitivity) 

A higher proportion of patients receiving DUT-TAM FDC had a MPR ≥ 0.80, ≥ 0.75, and ≥ 0.70 compared with AB/5ARI free-combination therapy and tamsulosin/finasteride combination therapy (all p < 0.0001; Table 4). The multivariate logistic regression analysis confirmed that DUT-TAM FDC therapy was associated with greater adherence compared with AB/5ARI free-combination therapy. Other factors associated with better adherence across all MPR categories were: age > 80 years, index prescription in 2013 or 2015, longer duration of prior AB treatment, and higher number of additional anatomical therapeutic chemical (ATC) classes (> 5 ATC classes versus ≤ 5 ATC classes) (Table 5). 

### End-of-therapy analysis 

Index therapy was re-started within 90 days of discontinuation by a small proportion of patients, 1.6% of DUT-TAM FDC-treated patients and 2.6% receiving AB/5ARI free-combination therapy. Therapy was changed within 90 days of discontinuation by 5.3% and 3.2% of patients, treated with DUT-TAM FDC and AB/5ARI free-combination therapy, respectively, and 76.9% of discontinued patients in the DUT-TAM FDC arm and 79.0% in the AB/5ARI free-combination arm had a confirmed therapy stop. 

## Discussion 

This retrospective, observational cohort study evaluated treatment persistence and adherence to DUT-TAM FDC compared with AB/5ARI free-combination therapy in over 140,000 patients with LUTS/BPH in Germany and was the first study to compare real-world levels of persistence and adherence to AB/5ARI (FDC or free combination) therapy in this patient group. 

Differences in persistence were generally small, irrespective of the length of gap period definition applied; however, statistically significant differences favoring DUT-TAM FDC or AB/5ARI free-combination therapy were present at various time points. Overall, persistence and adherence in both groups was low; however, differences in adherence were statistically significant in favor of the DUT-TAM FDC arm. 

A recent study conducted in the Netherlands by Drake et al. [23], where persistence was defined as a 30-day gap-period, a FDC of solifenacin and tamsulosin for LUTS/BPH (available in the Netherlands, but not in Germany) was shown to have significantly greater rates of persistence compared with free-combination therapy with these medications, or any combination of an anti-muscarinic medication and an AB. This remained true when the definition of time to discontinuation was increased to 45, 60, and 90 days [23]. After 12 months of therapy, persistence remained significantly greater in patients receiving an FDC compared with free-concomitant therapy [23]. Authors proposed that improved persistence in the FDC group could be partly explained by the convenience of taking 1 tablet as opposed to multiple doses [23]. Additionally, persistence with solifenacin in patients with LUTS/BPH is good compared with other anti-muscarinic therapies due to its favorable tolerability and efficacy profile, which may have acted as an incentive to continue treatment [23]. Analysis in the current study also shows a significantly greater persistence in patients receiving FDC compared with concomitant therapy at some time points; however, the magnitude of difference between treatment arms may not translate to meaningful clinical relevance. The study by Drake et al. assessed different drug classes from the current one and had a significantly smaller sample size. In comparison, the current study had a larger sample size, a longer follow-up period and minimally restrictive eligibility criteria, which helped to provide an accurate reflection of the real-world patient population as well as reducing the risk of obtaining inaccurate persistence and adherence data and optimizing generalizability. 

In the current study, differences in persistence between groups during the first 3 months could be partly explained by medication pack sizes. In Germany, DUT-TAM FDC is available in packs containing 30 or 90 capsules, with 90 capsules being the most commonly prescribed [GSK internal data]. Finasteride and tamsulosin, the most commonly prescribed free AB/5ARI combination [25], are available in regimens that include packs containing 30, 50, and 100 tablets; 100 tablets are the most commonly prescribed in Germany [25]. Our findings suggest that over 1 in 3 patients may discontinue therapy after just 1 prescription of either DUT-TAM FDC or AB-5ARI free combination. Given that 5ARI are disease-modifying treatments recommended when long-term treatment (≥ 12 months) is intended [9], this suggests a need for healthcare professionals to emphasize the importance of long-term treatment persistence to patients prior to commencing 5ARI combination therapy. In addition, between December 2015 and June 2016, there were several stock shortages for DUT-TAM FDC and for dutasteride monotherapy. Market data showed an increased switch during this time to tamsulosin and finasteride combination therapy, which may have impacted the MPRs for DUT-TAM FDC. 

In general, persistence was low in both groups. Despite a conservative definition of treatment discontinuation (90 days without prescription renewal), over 30% of patients in both groups discontinued AB plus 5ARI combination therapy, possibly after the first prescription. Less than 42% of patients were persistent for at least 12 months, the minimal treatment period required for a beneficial clinical effect of 5ARI therapy to be observed [6, 9], and less than 30% of patients were persistent 24 months after therapy initiation. Additionally, most patients who discontinued treatment in both therapy arms had a confirmed therapy stop date and only few restarted therapy or switched to a different AB and/or 5ARI, suggesting that treatment discontinuation was permanent. 

This low rate of persistence is consistent with findings in previous studies [6, 26]. In a study of 670 patients with LUTS/BPH, 12-month persistence with AB/5ARI (monotherapy or combination therapy) was only 36.6% [26]. The main reasons for discontinuation of treatment were resolved symptoms, failure of symptoms to improve and adverse events [26]. In addition to adequate income and a good patient-doctor relationship, disease-specific indicators of improved persistence were larger prostate volume and higher PSA [26], which are potential indicators of increased LUTS severity and the benefits of symptom control may have acted as an incentive for increased persistence. In the current study, factors associated with increased persistence included urologists as initial prescribers, age > 60 years, and longer duration of prior AB treatment, which are potentially indicative of more severe LUTS. With age-related disease progression and worsening LUTS, the benefits of symptom control may act as an incentive for treatment persistence and make patients more likely to tolerate any side effects [6]. 

A significantly higher percentage of patients receiving DUT-TAM FDC therapy were adherent during the current study compared with those receiving AB/5ARI in free combination; however, this may have a lesser impact on clinical outcomes due to low persistence overall. This reflects findings in previous studies, which have shown that adherence is better in medication regimens of 1 tablet per day, compared with 2 per day [18, 19, 20, 21]. A systematic review of the literature across many therapeutic areas concluded that the prescribed number of doses per day is inversely related to compliance [18], and multiple reviews show that patients with chronic diseases are more adherent with once-daily medication regimens than with more frequent doses [19, 20, 21]. These findings suggest that the reduction of AB/5ARI to a once-daily regimen may improve adherence in patients with LUTS/BPH, which may be of relevance to prescribing physicians in facilitating effective management of the condition. Nevertheless, in accordance with previous studies [6, 27], adherence in this study was found to be low overall. Factors associated with improved adherence included age > 80 years and longer duration of AB therapy, potentially indicative of more severe LUTS. In a retrospective study of 2,640 patients with LUTS/BPH, only 40% of patients were adherent to BPH medication, and treatment discontinuation was a strong predictor for BPH-related surgery [27]. Authors proposed that improved LUTS control may act as an incentive for improved adherence [27]. 

Poor persistence and adherence is associated with less favorable clinical outcomes; therefore, it is important for patients to commit to taking pharmacological therapy for LUTS/BPH as prescribed [6]. In chronic, long-term, non-life-threatening conditions such as BPH, the decision to persist with medical therapy primarily lies with patient perceptions of discomfort, symptom control, and inconvenience [6, 23]; unmet treatment expectations and poor tolerability may negatively impact the decision to persist [6, 28]. Counseling patients on realistic symptom improvement expectations and the importance of persistence in the long-term prevention of disease progression and risk of associated complications may be beneficial [6]. 

### Limitations 

There are several limitations of this study. Firstly, due to the large sample size, even small differences with potentially limited clinical relevance may have led to significant p-values and therefore should be interpreted with caution. No data was available to show whether patients took the medications according to the prescribed treatment regimen. Rather, it was assumed that if the medication was dispensed, it was correctly administered by the patient. However, regardless of whether all doses are correctly taken, differences between a once-daily regimen (DUT-TAM FDC) and a multiple-dose daily regimen (AB/5ARI free-combination therapy) cannot be discounted. Another limitation, which is common to retrospective observational studies using routinely collected electronic data platforms (not designed for specific research investigations), was the lack of availability of important variables in the IMS LRx (IQVIA) database such as diagnosis, severity of disease, and laboratory data (e.g., PSA).[24] Furthermore, dutasteride monotherapy was not reimbursed by statutory insurers in Germany until August 2017; therefore, data allowing the evaluation of dutasteride plus tamsulosin in combination as a best comparator to DUT-TAM FDC was limited. 

### Strengths 

As the IMS LRx (IQVIA) database collects anonymized patient histories and patient-level prescription data from pharmacy coding centers, patients were analyzed who actually had the medication in their hands (this allows to measure the medication possession ratio as an widely accepted surrogate for adherence) which eliminates the uncertainty of prescription data from medical records, where it is unknown if patients actually pick up their prescriptions in the pharmacy. 

Another strength of this study is the large sample size which may reduce the impact of the above-mentioned variables. 

Further, due to the retrospective design of the study the observed results may better reflect the “real-world” situation than more interventional study designs like controlled clinical trials or prospective observational trials. These approaches may use more sophisticated tools like medication event monitoring systems (medication bottles with an electronic cap counting every opening of the bottle) to reduce the uncertainty if patients actually take their medication, and maybe questionnaires for physicians and patients to further explore behavior and reasons for non-compliance. However, it is well known that such tools and study designs may influence the behavior of patients and thereby artificially increase persistence and adherence. 

## Conclusion 

The findings presented in this study indicate that persistence and adherence to AB/5ARI therapy in patients with LUTS/BPH in Germany is lower than DUT-TAM FDC overall. Differences in adherence were statistically significant in favor of the FDC once-daily regimen compared with the multiple-daily dosing combination therapy regimens. There were small differences in persistence between DUT-TAM FDC and AB/5ARI free-combination therapy over the 48-month period of evaluation; however, the magnitude of difference observed between treatment arms may not translate to meaningful clinical relevance. These findings may be useful clinically as improved adherence is associated with improved outcomes in patients with LUTS/BPH. Therefore, educating patients on the importance of persistence with medical therapy in long-term prevention of disease progression and its associated complications may be beneficial. Further studies are required to explore the key drivers for early treatment discontinuation and poor adherence to DUT-TAM FDC therapy in patients with LUTS/BPH. 

## Acknowledgment 

Medical writing support was provided by Gillian Wallace, MSc, and Lisa Auker, PhD, of Fishawack Indicia Ltd, UK, and funded by GSK. 

## Funding 

This study was funded by GlaxoSmithKline (GSK; study number 208854). 

## Conflict of interest 

Christoph Eisen, Michael Hennig, Burkay Adalig, Juan Manuel Palacios-Moreno, Vanessa Cortes and Zrinka Lulic are all employees of GSK and hold shareholder status in the company. Florian Gilg was an employee of GSK at the time of the study being performed. Karel Kostev is an employee of IQVIA. 

**Table 1.** Baseline demographics.

Variables	DUT-TAM FDC (n = 86,057)	AB/5ARI free combination (n = 55,610)	p-value
Prescriber specialty at index date, %			
Urologist	79.8	83.0	< 0.001
GP	15.4	14.7	< 0.001
Others	4.8	2.3	< 0.001
Age (mean, SD)	76.4 (9.3)	76.6 (9.3)	0.017
Age group, %			
≤ 60	6.0	5.7	0.037
61 – 70	18.8	18.9	0.789
71 – 80	40.3	39.9	0.175
> 80	34.9	35.4	0.028
Index year, %			
2011	9.6	7.8	< 0.001
2012	20.1	17.6	< 0.001
2013	18.4	16.4	< 0.001
2014	18.9	16.6	< 0.001
2015	17.4	19.1	< 0.001
2016	15.6	22.5	< 0.001
Prior treatment in BPH, %			
AB	66.5	66.1	0.131
5ARI	18.7	11.9	< 0.001
Therapy naïve	28.2	25.9	< 0.001
Duration of prior AB treatment, %			
Naïve	33.5	33.9	0.131
Up to 12 months	25.9	26.9	< 0.001
12 – 24 months	10.9	10.7	0.177
> 24 months	29.7	28.6	< 0.001
Max. ATC classes besides index therapy on a single day within 12 months prior to index date, %			
0	1.8	2.2	< 0.001
1 – 3	51.7	51.6	0.673
4 – 5	28.8	28.2	0.021
6 – 8	14.4	14.3	0.790
> 8	3.3	3.7	< 0.001

5ARI = 5-α reductase inhibitor; AB = α-blocker; ATC = anatomical therapeutic chemical; BPH = benign prostatic hyperplasia; DUT-TAM = dutasteride-tamsulosin; FDC = fixed-dose combination; GP = general practitioner; SD = standard deviation.

**Table 2.** Proportion of patients remaining persistent to therapy (90-day definition) at each time point.

Months since index date	Proportion persistent to therapy, % (p-value vs. DUT-TAM FDC)						
	DUT-TAM FDC	AB/5ARI free-combination therapy	Finasteride + tamsulosin	Finasteride + alfuzosin	Finasteride + terazosin	Finasteride + doxazosin	Finasteride + silodosin
6	58.7	60.8 (p < 0.0001)	61.2 (p < 0.0001)	59.7 (p = 0.1502)	59.8 (p = 0.4797)	61.3 (p = 0.1933)	52.4 (p < 0.0001)
12	41.8	41.0 (p < 0.0001)	41.4 (p = 0.0768)	39.4 (p < 0.0001)	40.1 (p = 0.2755)	44.0 (p = 0.2718)	34.0 (p < 0.0001)
18	33.4	32.3 (p < 0.0001)	32.7 (p = 0.0012)	29.9 (p < 0.0001)	33.4 (p = 1.0000)	35.4 (p = 0.2961)	25.3 (p < 0.0001)
24	28.2	27.1 (p < 0.0001)	27.5 (p = 0.0007)	24.5 (p < 0.0001)	29.9 (p = 0.2320)	29.9 (p = 0.3520)	21.3 (p < 0.0001)
36	21.3	21.0 (p = 0.0840)	21.4 (p = 0.5941)	18.6 (p < 0.0001)	22.8 (p = 0.2464)	23.8 (p = 0.1325)	16.0 (p < 0.0001)
48	16.6	17.0 (p = 0.0112)	17.3 (p < 0.0001)	14.9 (p = 0.0012)	19.2 (p = 0.0270)	18.6 (p = 0.1854)	12.5 (p < 0.0001)

5ARI = 5-α reductase inhibitor; AB = α-blocker; DUT-TAM = dutasteride-tamsulosin; FDC = fixed-dose combination.

**Table 3.** Factors affecting persistence to treatment for ≥ 24 months (logistic regression analysis).

Variable	90 days definition		60 days definition		30 days definition	
	Odds ratio	p-value	Odds ratio	p-value	Odds ratio	p-value
DUT-TAM FDC vs. AB/5ARI free-combination therapy	1.02	0.200	1.22	< 0.001	1.39	< 0.001
Prescriber specialty at index date: Urology	1.25	< 0.001	1.16	< 0.001	1.16	< 0.001
Prescriber specialty at index date: Others	1.04	0.455	1.03	0.572	1.05	0.485
Prescriber specialty at index date: GP	Reference		Reference		Reference	
Age ≤ 60	Reference		Reference		Reference	
Age 61 – 70	1.68	< 0.001	1.62	< 0.001	1.51	< 0.001
Age 71 – 80	2.08	< 0.001	2.00	< 0.001	1.83	< 0.001
Age > 80	2.00	< 0.001	1.91	< 0.001	1.76	< 0.001
Index year 2011	Reference		Reference		Reference	
Index year 2012	1.36	< 0.001	1.33	< 0.001	1.40	< 0.001
Index year 2013	1.41	< 0.001	1.38	< 0.001	1.50	< 0.001
Index year 2014	1.32	< 0.001	1.26	< 0.001	1.25	< 0.001
Index year 2015	1.46	< 0.001	1.36	< 0.001	1.37	< 0.001
Prior treatment with 5ARI	1.14	< 0.001	1.17	< 0.001	1.16	< 0.001
Duration of prior AB treatment: naïve	Reference		Reference		Reference	
Duration of prior AB treatment: ≤ 12 months	1.17	< 0.001	1.13	< 0.001	1.09	0.013
Duration of prior AB treatment: 12 – 24 months	1.46	< 0.001	1.34	< 0.001	1.27	< 0.001
Duration of prior AB treatment: > 24 months	1.65	< 0.001	1.60	< 0.001	1.51	< 0.001
Maximal number of ATC classes: 0	0.72	< 0.001	0.72	< 0.001	0.83	0.121
Maximal number of ATC classes: 1 – 3	1.05	0.327	1.10	0.081	1.14	0.064
Maximal number of ATC classes: 4 – 5	1.20	< 0.001	1.25	< 0.001	1.28	< 0.001
Maximal number of ATC classes: 6 – 8	1.18	< 0.001	1.17	0.004	1.15	0.057
Maximal number of ATC classes: > 8	Reference		Reference		Reference	

5ARI = 5-α reductase inhibitor; AB = α-blocker; ATC = anatomical therapeutic chemical; DUT-TAM = dutasteride-tamsulosin; FDC = fixed-dose combination; GP = general practitioner.

**Table 4.** Proportion of patients adherent to therapy.

Variable	DUT-TAM FDC	AB/5ARI free combination	χ^2^ vs. DUT-TAM FDC	Finasteride + tamsulosin	χ^2^ vs. DUT-TAM FDC
MPR ≥ 0.80, %	63.1	57.8	< 0.0001	58.8	< 0.0001
MPR ≥ 0.75, %	67.8	62.6	< 0.0001	63.5	< 0.0001
MPR ≥ 0.70, %	71.8	66.9	< 0.0001	67.6	< 0.0001

5ARI = 5-α reductase inhibitor; AB = α-blocker; DUT-TAM = dutasteride-tamsulosin; FDC = fixed-dose combination; MPR = medication possession ratio.

**Table 5.** Factors affecting adherence to treatment (logistic regression analysis).

Variable	MPR ≥ 0.80		MPR ≥ 0.75		MPR ≥ 0.70	
	Odds ratio	p-value	Odds ratio	p-value	Odds ratio	p-value
DUT-TAM FDC vs. AB/5ARI free combination therapy	1.28	< 0.001	1.29	< 0.001	1.30	< 0.001
Prescriber specialty at index date: Urology	1.01	0.621	1.02	0.212	1.03	0.166
Prescriber specialty at index date: Others	1.07	0.069	1.10	0.018	1.09	0.037
Prescriber specialty at index date: GP	Reference		Reference		Reference	
Age ≤ 60	Reference		Reference		Reference	
Age 61 – 70	1.01	0.651	1.03	0.399	1.02	0.573
Age 71 – 80	1.08	0.012	1.07	0.018	1.05	0.152
Age > 80	1.18	< 0.001	1.19	< 0.001	1.17	< 0.001
Index year 2011	Reference		Reference		Reference	
Index year 2012	0.96	0.109	0.96	0.123	0.98	0.359
Index year 2013	0.90	0.001	0.90	< 0.001	0.90	< 0.001
Index year 2014	0.99	0.572	0.99	0.687	0.99	0.628
Index year 2015	1.08	0.003	1.09	0.001	1.11	< 0.001
Prior treatment with 5ARI	0.95	0.008	0.97	0.071	0.97	0.131
Duration of prior AB treatment: naïve	Reference		Reference		Reference	
Duration of prior AB treatment: ≤ 12 months	1.06	< 0.001	1.07	< 0.001	1.09	< 0.001
Duration of prior AB treatment: 12 – 24 months	1.18	< 0.001	1.21	< 0.001	1.23	< 0.001
Duration of prior AB treatment: > 24 months	1.40	< 0.001	1.44	< 0.001	1.48	< 0.001
Maximal number of ATC classes: 0	0.53	< 0.001	0.52	< 0.001	0.52	< 0.001
Maximal number of ATC classes: 1 – 3	0.70	< 0.001	0.69	< 0.001	0.68	< 0.001
Maximal number of ATC classes: 4 – 5	0.89	0.002	0.90	0.004	0.89	0.003
Maximal number of ATC classes: 6 – 8	0.99	0.835	0.99	0.822	0.99	0.755
Maximal number of ATC classes: > 8	Reference		Reference		Reference	

5ARI = 5-α reductase inhibitor; AB = α-blocker; ATC = anatomical therapeutic chemical; DUT-TAM = dutasteride-tamsulosin; FDC = fixed-dose combination; GP = general practitioner; MPR = medication possession ratio.
